# Equilibrium Optimization-Based Ensemble CNN Framework for Breast Cancer Multiclass Classification Using Histopathological Image

**DOI:** 10.3390/diagnostics14192253

**Published:** 2024-10-09

**Authors:** Yasemin Çetin-Kaya

**Affiliations:** Department of Computer Engineering, Faculty of Engineering and Architecture, Tokat Gaziosmanpasa University, Tokat 60250, Turkey; yasemin.kaya@gop.edu.tr

**Keywords:** breast cancer classification, ensemble learning, equilibrium optimizer, histopathological image, deep learning

## Abstract

**Background**: Breast cancer is one of the most lethal cancers among women. Early detection and proper treatment reduce mortality rates. Histopathological images provide detailed information for diagnosing and staging breast cancer disease. **Methods**: The BreakHis dataset, which includes histopathological images, is used in this study. Medical images are prone to problems such as different textural backgrounds and overlapping cell structures, unbalanced class distribution, and insufficiently labeled data. In addition to these, the limitations of deep learning models in overfitting and insufficient feature extraction make it extremely difficult to obtain a high-performance model in this dataset. In this study, 20 state-of-the-art models are trained to diagnose eight types of breast cancer using the fine-tuning method. In addition, a comprehensive experimental study was conducted to determine the most successful new model, with 20 different custom models reported. As a result, we propose a novel model called MultiHisNet. **Results**: The most effective new model, which included a pointwise convolution layer, residual link, channel, and spatial attention module, achieved 94.69% accuracy in multi-class breast cancer classification. An ensemble model was created with the best-performing transfer learning and custom models obtained in the study, and model weights were determined with an Equilibrium Optimizer. The proposed ensemble model achieved 96.71% accuracy in eight-class breast cancer detection. **Conclusions**: The results show that the proposed model will support pathologists in successfully diagnosing breast cancer.

## 1. Introduction

Breast cancer (BC) is a type of cancer that can be fatal when cells multiply uncontrollably and form tumors. Over 2.3 million cases of breast cancer are diagnosed annually, making it the most prevalent cancer among adults. In 95% of countries, it ranks as the leading or second-leading cause of cancer-related deaths among women [1]. Early detection of the BC subtype is very important in terms of the treatment to be applied and the elimination of the risk of death. It can be detected at an early stage by experienced clinicians from various medical images, including ultrasonography, magnetic resonance imaging (MRI), and histopathological images.

Breast cancer types are classified into eight subgroups, each being either benign or malignant. Adenosis (A), fibroadenoma (FA), phyllodes tumor (PT), and tubular adenoma (TA) types are classified as benign and ductal carcinoma (DC), lobular carcinoma (LB), mucinous carcinoma (MC), and papillary carcinoma (PC) types are classified as malignant [2]. Manual classification of these various types by pathologists based on histopathological images can be problematic in terms of workload and risks that may arise as a result of incorrect decisions due to complex textural backgrounds and cell intertwining. Automatically detecting breast cancer types at an early stage using computer-aided systems ensures that the appropriate early treatment process begins immediately and prevents human-induced errors.

Convolutional neural networks (CNNs) are increasingly used to detect cancer and diseases in medical images [3,4]. CNNs, a type of deep learning, provide end-to-end automatic learning [5]. Despite their various architectures, they are primarily composed of convolution, pooling, and fully connected layers [6]. Certain issues arise when using CNNs to classify diseases from medical images. In medical images, the number of labeled data is generally limited, intra-class similarities are high, and class distributions are unbalanced [7,8]. For this reason, different CNN approaches, such as custom models, transfer learning, and ensemble learning, are used for classification purposes in medical images [9,10].

In the literature, transfer learning [11,12,13,14,15,16,17,18], scratch model [19,20], ensemble model [21,22,23], and vision transformer (ViT) [24,25] have been used in the detection of breast cancer. CNN models experience performance problems in medical images due to limited labeled datasets, intra-class similarities, and unbalanced class distributions. In the case of a limited dataset, CNN models also experience performance decreases in the test dataset due to over-learning problems during the training phase [26,27]. In addition to all these problems, successful feature extraction becomes quite difficult due to the overlapping of cells and complex textural backgrounds in BreakHis histopathological images. Furthermore, there are difficulties in distinguishing between the malignant tumor classes of ductal carcinoma and lobular carcinoma. The current custom model approach and transfer learning are insufficient in the classification of the BreakHis dataset.

In this study, an ensemble CNN model is proposed in which optimum weights are determined with the Equilibrium Optimizer (EO) algorithm to solve the existing problems. Performance results were obtained using 20 different state-of-the-art architectures with the transfer learning method. Due to the aforementioned issues, transfer learning results were insufficient to generate a high-performance model.

Custom models of various depths and widths were created to accomplish this. To avoid the vanishing gradient problem as the models grew deeper, residual connections were added to these custom models. Finally, channel attention was used to increase the weights of the important channels in the feature map, and spatial attention was used to increase the weights of the tumor-related important regions in the feature. By using pointwise and normal convolution together, the number of parameters is reduced, and more effective feature extraction is achieved. As a result, we propose a novel model called MultiHisNet. Both the proposed MultiHisNet model and state-of-the-art CNN models have been used to provide diversity in feature extraction. The optimum weights of the models were determined by equilibrium optimization. Thus, since the weights of the strengths of the models are increased, the existing problems are solved with ensemble learning.

The novelty and contributions of the study can be summarized in the following order.

- An original ensemble model based on CNN models is proposed for classifying eight breast cancer types.
- A novel CNN model called MultiHisNet is proposed to classify breast tumors.
- The results of the best 20 out of 110 proposed custom CNN models with different architectures are reported, and the behaviors of different architectures against problems in the BreakHis dataset are shown.
- Optimum ensemble model weights are determined by the EO algorithm.

The paper is divided into the following sections. The work conducted on the diagnosis of breast cancer is presented in Section 2. The study’s dataset and suggested framework are introduced in Section 3. The study’s findings and associated discussions are presented in Section 4. The final section presents the study’s conclusion as well as future research directions.

## 2. Background

Breast cancer is diagnosed using a variety of imaging techniques, including mammograms, ultrasound, magnetic resonance, and histopathologic images. Ultrasound and mammography imaging techniques detect areas of suspected cancer but do not aid in making a definitive diagnosis. Histopathologic images, on the other hand, provide detailed information at the cellular level, assisting in the detection of cancer, as well as its type and stage.

Researchers have conducted several studies to diagnose breast cancer using histopathological images and deep learning techniques [28]. Since the BreakHis dataset was created by Spanhol [29] and made publicly available to researchers, it has become one of the most frequently used datasets in breast cancer diagnosis studies with deep learning [28]. BreakHis is a dataset containing eight classes, four malignant and four benign. Table 1 presents a comparison of studies on the BreakHist dataset for breast cancer classification.

The first studies on this dataset used binary classification (benign and malignant). Spanhol et al. [12] performed transfer learning with the AlexNet model for breast cancer detection. Using sliding window and random extraction techniques, 32 × 32 and 64 × 64 patches were extracted from the dataset’s images and used during the training phase. An attempt to reduce the model’s complexity was performed by decreasing the size of the image sent to it. While this method was successful in binary classification at low magnifications, it failed at high magnifications. Garg and Singh [22] achieved binary classification accuracy ranging from 96.84% to 98.78% by combining the output of their proposed lightweight model with MobileNetV2 models using the ensemble technique. Zerouaoui et al. [30] performed feature extraction using seven different pretrained models and classified them binary for breast cancer detection with Decision Tree (DT), K-Nearest Neighbor (KNN), Multi-Layer Perceptron (MLP), and Support Vector Machine (SVM) classifiers. In the FNAC dataset, the best classification performance was obtained by using the DenseNet201 model and MLP, while in the BreakHis dataset, the most successful models were VGG16 at 100× magnification and DenseNet201 at other magnifications. Mewada et al. [20] proposed a CNN model that included spectral features generated through multi-resolution wavelet transform in addition to spatial features. Binary classification on the BerakHis dataset yielded an accuracy of 97.02% to 97.58%.

Han et al. [19] conducted one of the first studies to treat the BreakHis dataset as a multiclass classification. Data augmentation is used in this study to improve the training dataset. Furthermore, a deep learning architecture known as CSDCNN is proposed and trained in two different ways, with the results compared. The model is first trained from scratch, followed by transfer learning. In transfer learning, the model is trained using the ImageNet dataset before being fine-tuned with the BreakHis dataset. The proposed model achieves the best multiclass classification performance at the image level through transfer learning with data augmentation, with an average accuracy of 93.8%.

Transfer learning is another method for diagnosing breast cancer. Boumaraf et al. [11] conducted a transfer learning study using the ResNet-18 model. The last two residual blocks of the model and two newly added dense layers (128 and 8/2 neurons) were fine-tuned. Two approaches, magnification independent (MI) and magnification dependent (MD), were tested. In MI, images from four magnifications (40×, 100×, 200×, and 400×) in each class are collected in an experimental dataset and presented to the model, while in MD, four different models are trained using images from each magnification separately. While MI multiclass classification accuracy is 92.03%, this value is 98.42% in binary classification. The average accuracy in MD binary classification is 98.84%, while the average accuracy in multiclass classification is 92.15%.

Yari et al. [15] conducted a transfer learning study using the ResNet model. Two experimental datasets, with and without data augmentation, were used in the study. In MI, better classification performance was obtained with the augmented dataset in both binary and multiclass classification. MI multiclass classification yielded an accuracy of 94.33%. Vikrant et al. [13] compared the performance of the DenseNet, MobileNet, and ResNet models, as well as the softmax and SVM classifiers, in breast cancer diagnosis. The MobileNetV2 model and sigmoid/softmax classifiers had the highest success rate in MI classification on both multiclass (92%) and binary (97%) datasets. Mewada [17] improved the DenseNet161 model by adding residual layers and used it to detect breast cancer. The study found that using residual and special features resulted in 94.65% to 100% accuracy in binary classification and 96.76% to 97.59% accuracy in multiclass classification.

Zaalouk et al. [16] compared the performance of five different state-of-the-art models, namely DenseNet201, InceptionResNetV2, ResNet152, VGG19, and Xception, in breast cancer diagnosis. The transfer learning method was applied in two different ways. In the first method, only the fully connected layers of the models were trained, and the other layers were frozen, while in the second method, all layers were trained. The first method achieved 89.43% validation accuracy for binary classification and 69.68% validation accuracy for multiclass classification. In the second method, 98.92% and 93.29% validation accuracy were obtained for binary and multiclass classification, respectively. The Xception model outperformed all other multiclass classification models on the test dataset. MI and MD achieved accuracy rates of 93.32% and 90.22% to 97.01%, respectively. Xu et al. [14] proposed three models based on DenseNet121 and SENET architectures with different numbers and locations of SE modules. The best-performing model in multiclass classification is MFSCNet A, with accuracy values ranging from 94.36 to 98.41. Moreover, 400 images for each class were taken from the BreakHis dataset.

Ensemble model building is a technique used in breast cancer diagnosis research. Umer et al. [21] proposed a 35-layer CNN model and pre-trained it on the CIFAR-100 dataset. Then, using this trained model, features for breast cancer diagnosis were extracted (feature extraction), and the best features were selected using the PSO optimization algorithm. In addition, the RESNET-50 model was trained using transfer learning, and the features to be used in classification were determined by this model. The features extracted from these two models were combined and classified using machine learning classifiers. The BreakHis dataset yielded the highest accuracy of 90.10% ensemble subspace KNN machine learning algorithm in the eight-class classification.

ViT is another technique used to diagnose breast cancer. He et al. [24] proposed the Deconv-Transformer model, which focuses on the staining feature of histopathological images. The proposed model first combines the RGB and HED color spaces of the images before transmitting them to the vision transformer. The highest average accuracy achieved in binary classification is 93.02. Tummala et al. [25] used Swin Transformers to diagnose breast cancer. Four different models were trained: base, large, tiny, and small, which were then combined using the average ensemble learning technique. With the ensemble model, an accuracy of 93.4% was obtained in the MI multiclass classification, while accuracy values in the range of 92.6–96.0% were obtained in the MD multiclass classification.

Long Short-Term Memory (LSTM) architecture is another deep learning technique used in breast cancer diagnosis. Srikantamurthy et al. [31] proposed a combination of CNN (transfer learning with pretrained models) and LSTM architectures. The proposed CNN-LSTM model achieves an accuracy score of 99% in binary classification and 92.5% in eight-class classification.

An analysis of the literature demonstrates that training models with an excessive number of parameters may result in overfitting. A limited variety of features is obtained when a single model is used in transfer learning models. Furthermore, models like ViT that need a lot of images for training might not be able to use the BreakHis dataset. The proposed ensemble models are usually performed by the averaging method. Since all models are combined with the same weight, the strengths of the models are not revealed. As a result, more advanced models and ensemble techniques should be used in the BreakHis dataset, including unbalanced class distribution and high intra-class similarity.

## 3. Materials and Methods

### 3.1. Dataset

**Figure 1:**
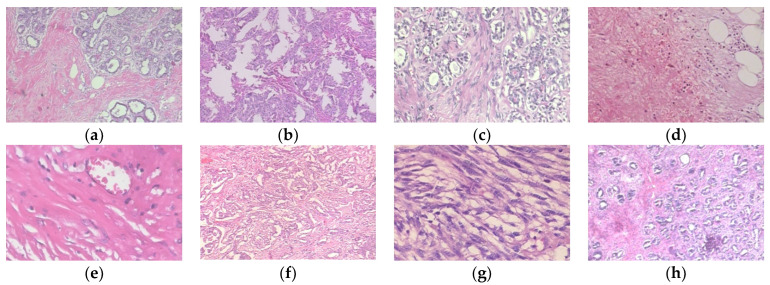
Sample images of the classes in the BreakHis dataset. (**a**) A(40×); (**b**) DC(40×); (**c**) F(100×); (**d**) LC(100×); (**e**) MC(200×); (**f**) PC(40×); (**g**) PT(200×); (**h**) TA(40×).

BreakHis dataset [29] consists of 7909 images from 82 patients using different magnification factors (40×, 100×, 200×, and 400×) used in the study. Adenosis (A), ductal carcinoma (DC), fibroadenoma (F), lobular carcinoma (LC), mucinous carcinoma (MC), papillary carcinoma (PC), phyllodes tumor (PT), and tubular adenoma (TA) are among the eight classes included in the dataset. DC, LC, MC, and PC are malignant classes, whereas A, F, PT, and TA are benign. Sample images of the classes are presented in Figure 1.

In the study, 80% of the images in the dataset were used for training and 20% for testing. In addition, 10% of the training dataset was used for validation. The distribution of images belonging to the classes in the dataset used in the study is presented in Table 2.

Since the classes in the dataset do not contain an equal number of images, they have an unbalanced distribution. In order to solve the problems caused by unbalanced distribution, such as overfitting to the class with a large number of examples, not obtaining information about the class with a small number of examples, and difficulty in recognition, class weight was used [32,33]. The formula used to calculate class weights for classes is shown in Equation (1). In the equation, *n* represents the number of classes, *N_i_* represents the number of images in class *i*, and *N_k_* represents the number of images in each class (*k*). The dataset’s class weights for A, DC, F, LC, MC, PC, PT, PT, and TA are 2.23, 0.29, 0.98, 1.58, 1.25, 1.77, and 2.18, respectively.

(1) $${Classweight}_{i}=\frac{\sum_{k=1}^{n}N_{k}}{n\times N_{i}}$$

### 3.2. Proposed Framework

**Figure 2:**
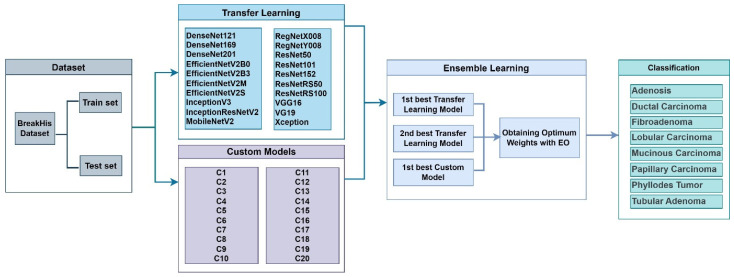
Proposed framework.

The framework proposed in the study is presented in Figure 2. In the first phase of the study, the dataset was prepared. The images in the BreakHis dataset were divided into training and test folders to be suitable for magnification-independent multiclass realization. In the second stage, transfer learning was performed with 20 different state-of-the-art models (see Figure 2) that have shown successful performance with ImageNet dataset in the literature. In the third stage, extensive experimental studies were carried out to determine the custom models that perform successfully for breast cancer diagnosis. The performances of convolution, pointwise convolution, dense layers, residual connectivity, and attention modules with different numbers and sizes were analyzed. In the fourth stage, the ensemble model was created. The ensemble model contained the best-performing transfer learning and custom models. By determining the weights of the models using EO, it was possible to demonstrate the impact of the models’ strong features on the outcome.

CNN models were constructed and trained using the deep learning packages TensorFlow and Keras. The computations and procedures were carried out on a typical PC setup with an Intel i5-8400 CPU, an NVIDIA GeForce GTX 1080 Ti GPU with 11 GB of RAM, and 16 GB of RAM.

### 3.3. Transfer Learning

At this stage, transfer learning was performed with 20 state-of-the-art models (see Figure 2. The fully connected layers of the models were removed, and then two dense layers and a classification layer with eight neurons were added to the models. Horizontal and vertical flip data augmentation techniques were applied to the dataset during the training phase. All layers of the models were included in the training, and no layers were frozen. The models were trained for 100 epochs. The learning rate used was 0.0001, the batch size was 16, and the activation function was Relu. The image size sent to the models is 224 pixels.

GridSearch optimization was used to determine the hyperparameters. The hyperparameter ranges were determined after reviewing the literature and previous studies. Table 3 shows the hyperparameter ranges and the best-performing hyperparameters in grid search.

### 3.4. Proposed Custom Model Development

In the proposed custom model development stage, three different structures were created. The first structure tested convolutional blocks with varying numbers of filters as well as dense models with varying numbers of neurons. The second structure included pointwise convolution layers and residual connections in the models. The third structure included channel and spatial attention modules in the models. To determine the best-performing custom model for breast cancer diagnosis, 110 models were created, and 20 of them were chosen to demonstrate different features and were reported. Model architectures are presented in Table 4 and Table 5. The models include a MaxPooling (MP) layer after each convolution block. There is also BatchNormalization after each convolution layer.

Models C1–C7 in the first structure have varying numbers of filters, convolution layers, dropout rates, and neurons in dense layers. The C8-C10 models were enhanced with residual connections and a pointwise convolution layer in addition to convolutional layers.

Point-wise convolution layer: When we increase the depth of the model, the number of parameters also increases. This leads to an increase in computational power. To address this issue, we used 1 ∗ 1 convolutional layers. The literature refers to these 1 ∗ 1 structures as pointwise convolution. Using pointwise convolution before the standard convolution layer reduces the number of channels in the input feature map [34]. This significantly reduces both the computational cost and the number of parameters. Moreover, even though pointwise convolution reduces the number of parameters, the model is still capable of extracting significant features. After the pointwise convolution, the normal convolution can concentrate on spatial relationships by picking and integrating key features across channels [35,36]. The model is less likely to overfit and is able to concentrate more on significant features when the dimensionality is reduced prior to applying a normal convolution.

Residual connection: In the study, residual connections were introduced into the convolution blocks. These connections are intended to prevent the vanishing gradient problem [37]. ResNet [38] architecture implements residual connection blocks through a merge with add operation. In our work, however, we use the concatenate operation on convolution blocks similar to the DenseNet [39] architecture. The output of one block is passed on to the next block by adding the output of the previous block.

In addition to the pointwise convolution layer and residual connections, the C11-20 models include attention modules. The study used the channel and spatial attention module as the attention module, which was added as the final element of each convolution block.

Channel and spatial attention module: Attention modules highlight key traits while suppressing uninteresting ones [40]. When detecting disease using medical imaging, it is critical to recognize lesions or patterns. Cancerous lesions may be situated in a specific region of the image and are difficult to differentiate from the surrounding area. In this situation, attention processes identify and highlight these critical areas, assisting in the detection and diagnosis of problems. Another crucial benefit of attention mechanisms is the capacity of attention maps to shed light on a model’s logic behind a specific choice. Interpretability is important in medical applications because physicians need to understand the underlying assumptions that underlie a model’s diagnosis or prescription. Spatial attention focuses on “where” beneficial information is found, and channel attention focuses on “what” is essential when given with an input image [41].

Custom models were trained for 100 epochs. In the training phase, data augmentation was applied to the dataset using horizontal and vertical flip techniques. The learning rate started with 0.001 at the beginning of the training and was observed for 10 epochs, and if there was no improvement in accuracy, it was decreased by 0.1. In this way, it was finally reduced to 10^−5^. The batch size used in the study is 16, the optimization algorithm is Adam, and the image size is 224 pixels.

### 3.5. Ensemble Model

In the study, after the transfer learning and custom models were trained, the two most successful models were chosen to form an ensemble model. Ensemble models were used to create an integrated model that combines the strengths of the models for improved performance.

When the ensemble model makes a prediction for an image, it collects predictions for that image from all the models in the ensemble. Various voting techniques are used to generate results based on these predictions [42]. The first one is majority voting. The prediction produced by the highest number of models becomes the prediction of the ensemble model.

In average voting, the result of the ensemble model is generated by averaging the predictions. The most significant disadvantage of these techniques is that each model has an equal impact on the outcome. However, the success of the ensemble model can be improved by increasing the influence of the models that perform better in a classification decision on the outcome. At this point, it is critical to determine the models’ weights. The weighted voting technique assigns weights to the models, which influence the ensemble model’s prediction decision based on these weights. In our study, the EO algorithm was used to calculate these weights.

#### Equilibrium Optimizer

The EO algorithm is a population-based meta-heuristic approach that draws inspiration from nature [43,44]. Similar to the particle swarm optimization (PSO) algorithm, the EO algorithm begins with a randomly generated initial population. Each particle or solution element in the initial population has a solution set for the problem of interest. Each particle in the initial population is trained according to the fitness function, and fitness values are obtained. In this problem, the fitness function is the summation of the prediction probabilities of each model according to the weight ratio in the particles and the calculation of the accuracy value according to the final probabilities. As in other meta-heuristics algorithms, the next positions of each particle in the solution space are calculated according to a certain mathematical model, local search capacity, and global search capability. An iteration is completed after updating the new positions of each particle in the solution space.

When determining the optimal weight of each model in the EO algorithm, the corresponding accuracy value is calculated for each particle value. The EO algorithm selects the first four particles for the Equilibrium pool by ranking the accuracy value of the particles from largest to smallest. A particle containing the average values of these four values is also added to the Equilibrium pool. The main goal here is to determine the next position of a particle by randomly selecting a particle from the Equilibrium pool and updating it according to the exploration and exploration approaches. Exploitation is when a particle searches for solutions around the current best, and exploration is when it searches for solutions in different regions of the solution space. In the EO algorithm, the position of each particle is updated throughout the iterations using Equation (2).

(2) $$C=C_{eq}+\left(C-C_{eq}\right)F+\frac{G}{\lambda V}\left(1-F\right)$$

**Figure 3:**
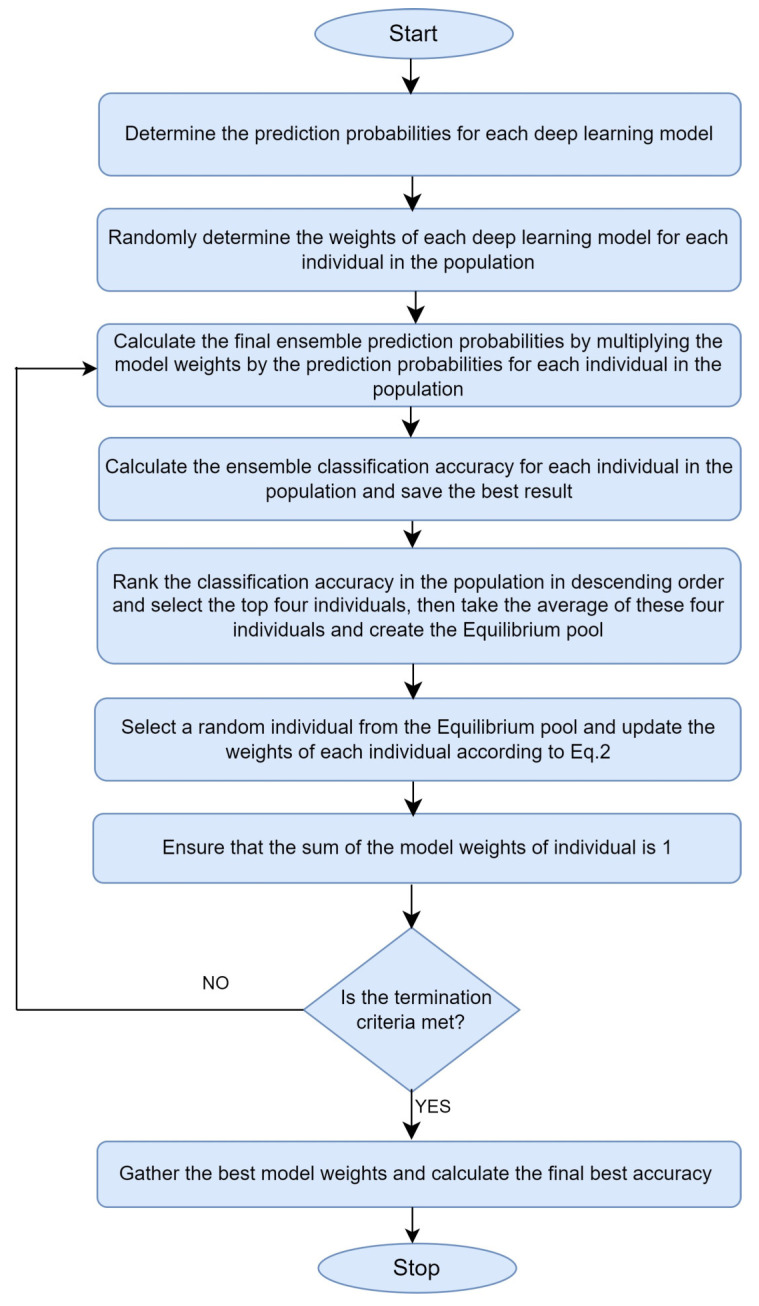
EO algorithm flow diagram.

In Equation (2), we denote the position of each particle in the population by *C*. Here, Ceq is randomly selected from the Equilibrium pool to update each position *C*. The difference between particle *Ci* and *Ceq* is multiplied by an exponential term (*F*). The term *F* tries to balance between local and global search and is an exponential function with random terms. *V* is a constant that usually takes the value 1 or 2. *λ* is a random value between 0 and 1. *G* is the Generation rate, which is also determined by a random number. Our population size is 30, and the optimization algorithm runs for 1000 iterations. Figure 3 represents the flow diagram of the EO algorithm used in the study.

In the tests performed by Faramarzi et al. [43], the EO algorithm outperformed several optimization algorithms, such as Genetic Algorithm (GA), Grey Wolf Optimizer (GWO), and particle swarm optimization (PSO). The global search of EO was found to be better. EO algorithm has been successfully used in optimization studies in different fields, such as image segmentation and model hyperparameter optimization in disease classification [45]. Considering these features, we preferred to use EO algorithm in our study.

In the EO-based ensemble optimization proposed in this study, EO is first used to assign weights to the models. The models then provide class predictions, and their weighted average is computed. The class with the highest probability is chosen as the ensemble model’s final prediction.

In fact, in population-based optimization algorithms such as PSO, ABC, Ant colony, EO, and Genetic Algorithm, each individual or element in the population represents a solution, and these solutions take random values between 0 and 1 in the initial phase. In other words, each solution element in the population starts with random values and continues with new values in the following iterations by updating each solution element under certain randomness conditions with methodological approaches inspired by nature. The biggest disadvantage of algorithms such as grid search and random search is that they do not take into account the solution values found in previous iterations. Population-based algorithms such as EO, on the other hand, take into account the solutions found in previous iterations and perform a balanced search around the best solution in the solution space so far (local search—exploitation) and in different areas of the solution space (global search—exploration). Considering the values of the best solution elements in the previous iterations, the search for new solution spaces in the direction of exploitation and exploration ensures that the best result is reached in the shortest time.

## 4. Results and Discussion

### 4.1. Results of Transfer Learning

In Table 6, the test performances of 20 state-of-the-art models trained with transfer learning are reported in terms of accuracy, precision, recall, and F1-score metrics. The highest accuracy value is 93.68% with the RegNetX008 model, while the second-best value is 93.49% with the DenseNet169 model. DenseNet201 and RegNetYoo8 models achieved the third highest value with an accuracy of 93.11%. The F1-score value, which represents the harmonic mean of precision and recall values, was highest for the RegNetX008 (93.19%) and DenseNet169 (92.95%) models.

### 4.2. Results of Custom Model

Table 7 presents the test performance metrics obtained using custom CNN models. Among the models created with basic convolutional blocks, the C7 model performed the best, with 92.98% accuracy and a 92.33% F1 score. The C10 model with residual blocks yielded the highest accuracy (93.62%) and F1 score (93.19%). This demonstrates the beneficial effects of residual blocks on breast cancer detection performance.

Table 8 shows the performance metrics for the models, which include attention blocks (links). With these models, accuracy ranged from 93.05% to 94.69%. The C20 model has the highest accuracy value (94.69%), followed by the C19 model (94.37%) and the C18 model (94.31%). In the C18 model, the channel and spatial attention module is added after each convolutional block, whereas in the C20 and C19 models, the output is concatenated with the previous block’s output and presented as input to the next block, in addition to CNN, channel and spatial attention module. This had a positive effect on performance. In the rest of the study, the best-performing C20 model will be referred to as the MultiHisNet model.

**Figure 4:**
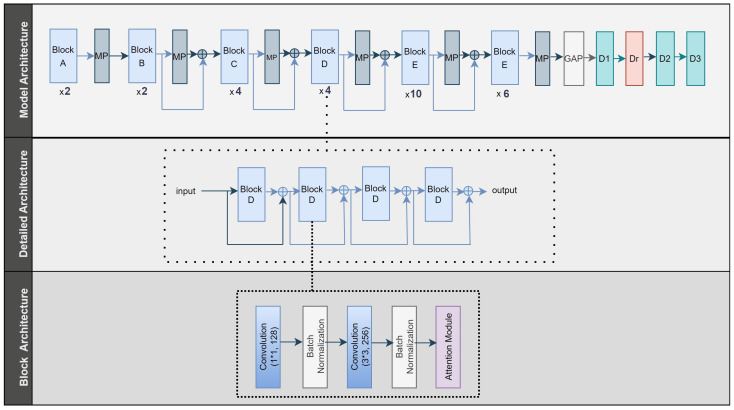
The architecture of the proposed MultiHisNet model.

Figure 4 depicts the architecture of the MultiHisNet model. The model has five block types (Blocks A-E). The model consists of 28 blocks (2 Block A, 2 Block B, 4 Block C, 4 Block D, 10 Block E, and 6 Block E), with a MaxPooling (MP) layer between them. Starting with Block B, the output of each convolutional block is concatenated with the output of the previous block and fed into the next layer. The GlobalAveragePooling (GAP) layer connects the dense (D) layers. The model consists of three dense layers, each with 512, 18, and 8 neurons. Following the first dense layer, dropout (Dr) was applied at a rate of 0.4. The blocks contain two convolution layers, followed by the BatchNormalization layer and, finally, the channel and spatial attention module. Table 9 shows the various filter size and number combinations used in each block’s convolution layers.

### 4.3. Detailed Analysis of the Best-Performing Models

**Figure 5:**
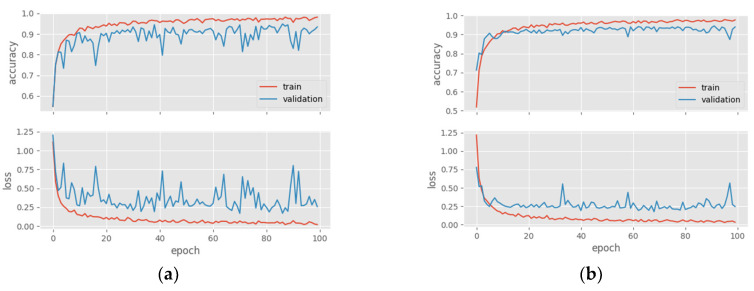

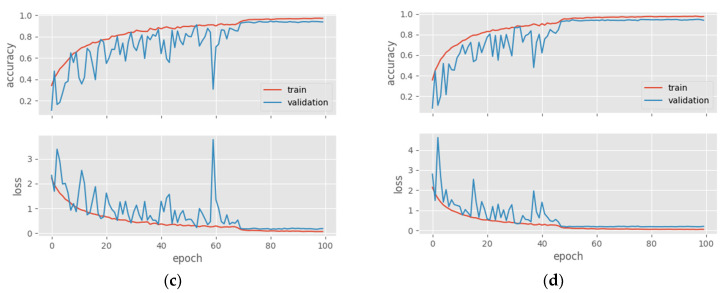
Train/validation accuracy and loss graphs of models in ensemble learning. (**a**) DenseNet169; (**b**) RegNetX008; (**c**) MultiHisNet; (**d**) 2nd-Best Custom Model (C19).

The training/validation and test outcomes of the top-performing transfer learning and custom models are thoroughly reviewed in this subsection. Figure 5 shows the accuracy and loss graphs of the two best-performing models from transfer learning and custom models. When the graphs of the RegnetX008 model are analyzed, slight overfitting is observed, while in the DenseNet169 model, although the train and validation curves follow each other, the validation curve is lower.

**Figure 6:**
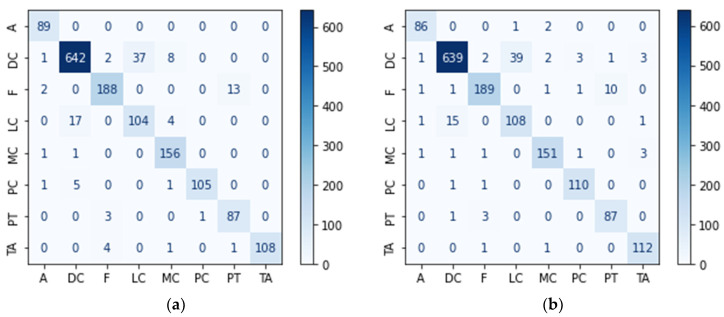

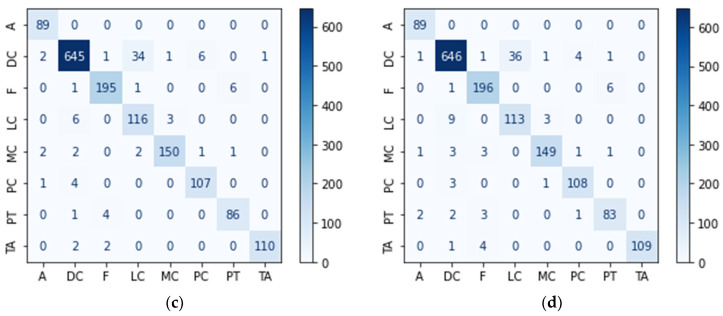
Confusion matrices of models in ensemble learning. (**a**) DenseNet169; (**b**) RegNetX008; (**c**) MultiHisNet; (**d**) 2nd-Best Custom Model (C19).

Figure 6 depicts the confusion matrices for the two best models from the transfer learning and custom models. The *x*-axis displays the predicted labels, while the *y*-axis displays the true labels. When analyzed on a class basis, three models correctly classified 89 images from the A class (see Figure 6a,c,d), while the RegNetX008 model made a small number of incorrect predictions (3 errors). When we look at the DC class, we notice that it has the highest number of incorrect predictions across all models. When the incorrect predictions were examined, it was discovered that the LC class was frequently predicted incorrectly. It was observed that custom models produced more successful predictions than transfer learning models in DC, F, and LC classes. While DenseNet169 was the most successful model in MC class prediction, RegNetX008 was the most successful model in PC and TA classes.

These results show that the performances of different models are also different on a class basis. The class predicted by each model with high accuracy may differ. This confirms our decision to create a more effective model that feeds on differences by combining multiple models with ensemble learning in the framework proposed in the study.

### 4.4. Results of the Ensemble Model

For use in ensemble models, two models with the best classification performance were selected from custom models and transfer learning models. The selected models are presented in Table 10.

According to the proposed framework, the ensemble model was generated by integrating the two best transfer learning models (RegNetX008 and DenseNet169) with the best custom (MultiHisNet) models. EO computed the following weights for the models: 0.50 for MultiHisNet, 0.32 for DenseNet169, and 0.18 for RegNetX008. The proposed ensemble model produced an accuracy of 96.71%.

Additionally, three more ensemble models (E1–E3) were created to examine the ensemble performances of various models, and the results are shown in Table 11. When the two best custom models (C1 and C2) were combined with transfer learning models, E1 and E2 achieved an accuracy of 96.46%. When the best two transfer learning models (T1 and T2) were combined with the second-best custom model (C2), the accuracy of E3 was 96.52. The ensemble model proposed in the study outperformed all three ensemble models.

### 4.5. Discussion

#### 4.5.1. Comparison of the Models

Table 12 presents the assessment of the suggested custom models. Models using only CNN blocks produced the highest accuracy of 92.98. The classification success rose to 93.62 when residual connections were added to CNN blocks. Later, the classification success increased even more when channel and spatial attention modules were added to the models. An accuracy of 96.71% was attained in the best classification performance when the models were combined with ensemble learning.

#### 4.5.2. Comparison with Similar Studies

To compare our findings to similar studies in the literature, we examined studies that used the BreakHis dataset for breast cancer diagnosis and performed MI multi-class classification. Table 13 summarizes studies that used the BreakHis dataset for eight-class classification. The proposed ensemble model is more accurate than all the studies in Table 13.

When the studies are examined in terms of the methods used, it is discovered that transfer learning and ensemble learning are employed. Transfer learning was the most preferred method among researchers. The success rate of models using the transfer learning method ranges between 92% and 94.30%. Models used for transfer learning contain a large number of parameters, and overfitting problems are encountered in models with a small amount of data.

When investigating ensemble models, it was discovered that two techniques were used: model and feature ensemble. Umer et al. [21] used feature ensembles in their study. The features obtained from custom and transfer learning models were first reduced, then ensembled and classified using machine learning algorithms. Tummala et al. [25] achieved a 93.4% accuracy rate by combining four SwinT models. Since vision transformers require a large amount of data to train, they struggle to achieve high performance in medical datasets with fewer images. Wang et al. [46] added attention modules to the VGG16 and ResNet-50 models. The ensemble model created using the soft voting technique achieved a multi-class classification accuracy of 94.11%.

The proposed ensemble model in the study includes both the custom (MultiHisNet) model and the transfer learning (DenseNet169, RegNetX008) models. The proposed ensemble model achieved 96.71% accuracy in multi-class breast cancer diagnosis, outperforming the ensemble model studies listed in Table 13. The main factors in this success are the successful custom model developed through extensive experimental studies and the EO used to determine the ensemble model weights. While the MultiHisNet produces more successful models with fewer parameters and more effective features due to the pointwise convolution layers it contains, it also avoids the vanishing gradient problem with residual connections and knows what and where to focus on the image through to the channel and spatial attention module. Furthermore, rather than giving equal influence to all models in the ensemble model, weights were assigned to the models that would be included in the ensemble model with EO. As a result, more successful classification decisions could be made by leveraging the models’ strengths.

## 5. Conclusions

Breast cancer is one of the most common and deadly forms of cancer in women. When patients are diagnosed early and correctly, their life expectancy and quality of life improve as a result of effective treatment programs. Histopathological images, which provide detailed information at the cellular level, can also be used to determine the type and severity of the disease in breast cancer detection. This study used 20 state-of-the-art models for transfer learning. In addition, 20 different custom CNN models were evaluated for breast cancer detection. As a result, we propose a novel CNN model called MultiHisNet to classify breast tumors. The MultiHisNet model has fewer parameters, contains pointwise convolution layers, residual connections, and channel and spatial attention modules, is free of overfitting and vanishing gradient problems, and knows which region and structure to focus on in the image.

The ensemble model, which included the most successful custom and transfer learning models, was built with weights optimized using EO. The proposed ensemble model achieved 96.71% accuracy in multi-class classification for breast cancer diagnosis. In future studies, studies are planned to be conducted to improve the detection of the LC class, which has lower accuracy on a class basis. In addition, it is planned to detect tumor regions with a segmentation study, and models will be trained using the images.

## Figures and Tables

**Table 1:** Comparison of the related studies.

Reference	Method	Classification	Magnification	Performance (Accuracy %)
Spanhol et al. [12]	Transfer Learning	Binary	MD	80.8–85.6%
Zerouaoui et al. [30]	Feature Extraction + ML algorithms	Binary	MD	91.73–93.93%
Mewada et al. [20]	Custom CNN	Binary	MD	97.02–97.58%
Garg and Singh [22]	Ensemble Model	Binary	MD	96.84–98.78%
Han et al. [19]	Custom CNN + transfer learning	Binary	MD	92.9–96.9%
		Multiclass	MD	92.8–93.9%
Boumaraf et al. [11]	Transfer Learning	Binary	MD	98.84%
			MI	98.42%
		Multiclass	MD	92.15%
			MI	92.03%
Yari et al. [15]	Transfer Learning	Binary	MD	97.12–99.05%
			MI	99.01%
		Multiclass	MD	94.23–97.96%
			MI	94.33%
Vikranth et al. [13]	Transfer Learning	Binary	MD	98%
			MI	97%
		Multiclass	MD	86–91%
			MI	92%
Zaalouk et al. [16]	Transfer Learning	Binary	MD	99.42–100%
			MI	98.99%
		Multiclass	MD	90.22–97.01%
			MI	93.32%
Xu et al. [14]	Transfer Learning	Binary	MD	99.05–99.89%
		Multiclass	MD	94.36–98.41%
Mewada [17]	Transfer Learning	Binary	MD	94.65–100%
		Multiclass	MD	96.76–97.59%
Umer et al. [21]	Ensemble Model (6 B-Net)	Multiclass	MI	90.1%
He et al. [24]	Transformers	Binary	MI	93.02%
Tummala et al. [25]	Ensemble of SwinT	Binary	MI	99.6%
		Multiclass	MD	92.6–96.0%
			MI	93.4%
Srikantamurthy et al. [31]	CNN + LSTM	Binary	MD	98.07–99.75%
		Multiclass	MD	88.04–96.3%

**Table 2:** Distribution of the dataset.

Class	Train	Test	Total
Adenosis (A)	355	89	444
Ductal Carcinoma (DC)	2761	690	3451
Fibroadenoma (F)	811	203	1014
Lobular Carcinoma (LC)	501	125	626
Mucinous Carcinoma (MC)	634	158	792
Papillary Carcinoma (PC)	448	112	560
Phyllodes Tumor (PT)	362	91	453
Tubular Adenoma (TA)	455	114	569
Total (classes)	6327	1582	7909

**Table 3:** Hyperparameters.

Hyperparameter	Range	Best
Dense Layer 1	128, 256, 512, 1024, 2048	512
Dense Layer 2	128, 256, 512, 1024, 2048	256
Dropout	0.2, 0.3, 0.4, 0.5	0.2
Optimization algorithm	Adam, SGDNesterov	Adam

**Table 4:** Architecture of the custom models that contain CNN layers and residual connections.

	C1	C2	C3	C4	C5	C6	C7	C8	C9	C10
CNN Block1	$\left[3x3,\;16\right]\times 1$	$\left[3x3,\;16\right]\times 1$	$\left[3x3,\;16\right]\times 2$	$\left[3x3,\;16\right]\times 2$	$\left[3x3,\;16\right]\times 2$	$\left[3x3,\;16\right]\times 2$	$\left[3x3,\;16\right]\times 2$	$\left[3x3,\;16\right]\times 2$	$\left[3x3,\;16\right]\times 2$	$\left[3x3,\;16\right]\times 2$
CNN Blok2	$\left[3x3,\;32\right]\times 2$	$\left[3x3,\;32\right]\times 2$	$\left[3x3,\;32\right]\times 2$	$\left[3x3,\;32\right]\times 2$	$\left[3x3,\;32\right]\times 2$	$\left[3x3,\;32\right]\times 2$	$\left[3x3,\;32\right]\times 2$	$\left[3x3,\;32\right]\times 2$	$\left[3x3,\;32\right]\times 2$	$\left[\begin{array}{c} 3x3,\;32\times 1\;\\ 3x3,\;128\times 1 \end{array}\right]$
CNN Block3	$\left[3x3,\;64\right]\times 2$	$\left[3x3,\;64\right]\times 2$	$\left[3x3,\;64\right]\times 2$	$\left[3x3,\;64\right]\times 2$	$\left[3x3,\;64\right]\times 2$	$\left[3x3,\;64\right]\times 2$	$\left[3x3,\;64\right]\times 2$	$\left[3x3,\;64\right]\times 2$	$\left[3x3,\;64\right]\times 2$	$\left[\begin{array}{c} 1x1,\;64\times 1\;\\ 3x3,\;64\times 1\\ 3x3,\;256\times 1 \end{array}\right]$
CNN Block4	$\left[3x3,\;128\right]\times 2$	$\left[3x3,\;128\right]\times 2$	$\left[3x3,\;128\right]\times 2$	$\left[3x3,\;128\right]\times 2$	$\left[3x3,\;128\right]\times 2$	$\left[3x3,\;128\right]\times 2$	$\left[3x3,\;128\right]\times 2$	$\left[3x3,\;128\right]\times 2$	$\left[3x3,\;128\right]\times 2$	$\left[\begin{array}{c} 1x1,\;128\times 1\;\\ 3x3,\;128\times 1\\ 3x3,\;512\times 1 \end{array}\right]$
CNN Block5	$\left[3x3,\;256\right]\times 3$	$\left[3x3,\;256\right]\times 3$	$\left[3x3,\;256\right]\times 2$	$\left[\begin{array}{c} 3x3,\;256\times 2\\ 3x3,\;512\times 2 \end{array}\right]$	$\left[3x3,\;256\right]\times 3$	$\left[\begin{array}{c} 3x3,\;256\times 2\;\\ 3x3,\;512\times 1 \end{array}\right]\;$	$\left[\begin{array}{c} 3x3,\;256\times 1\;\\ 3x3,\;512\times 2 \end{array}\right]\;$	$\left[3x3,\;256\right]\times 2$	$\left[3x3,\;256\right]\times 3$	$\left[\begin{array}{c} 1x1,\;256\times 1\;\\ 3x3,\;256\times 2\\ 3x3,\;512\times 1 \end{array}\right]$
CNN Block6	-	-	$\left[3x3,\;512\right]\times 2$	-	-	-	-	$\left[3x3,\;256\right]\times 2$	$\left[\begin{array}{c} 3x3,\;256\times 1\;\\ 3x3,\;512\times 2 \end{array}\right]$	$\left[\begin{array}{c} 1x1,\;256\times 1\;\\ 3x3,\;256\times 1\\ 3x3,\;512\times 2 \end{array}\right]$
CNN Block7	-	-	-	-	-	-	-	$\left[3x3,\;512\right]\times 3$	-	-
	Flatten	GAP	GAP	GAP	GAP	GAP	GAP	GAP	GAP	GAP
Dense1	512	512	512	512	1024	512	512	512	512	512
Dropout	0.2	0.2	0.2	0.2	0.2	0.3	0.2	0.4	0.2	0.2
Dense2	256	256	256	256	256	128	128	128	96	128

**Table 5:** Architecture of the custom models that contain attention modules.

	C11	C12	C13	C14	C15	C16	C17	C18	C19	C20
CNN Block1	$\left[3x3,\;32\right]\times 2$	$\left[3x3,\;32\right]\times 2$	$\left[3x3,\;32\right]\times 2$	$\left[3x3,\;32\right]\times 2$	$\left[3x3,\;32\right]\times 2$	$\left[3x3,\;32\right]\times 2$	$\left[3x3,\;32\right]\times 2$	$\left[3x3,\;32\right]\times 2$	$\left[3x3,\;32\right]\times 2$	$\left[3x3,\;32\right]\times 2$
CNN Blok2	$\left[\begin{array}{c} 1x1,\;32\\ 3x3,\;64 \end{array}\right]\times 2$	$\left[\begin{array}{c} 1x1,\;32\\ 3x3,\;64 \end{array}\right]\times 2$	$\left[\begin{array}{c} 1x1,\;32\\ 3x3,\;64 \end{array}\right]\times 3$	$\left[\begin{array}{c} 1x1,\;32\\ 3x3,\;64 \end{array}\right]\times 2$	$\left[\begin{array}{c} 1x1,\;32\\ 3x3,\;64 \end{array}\right]\times 2$	$\left[\begin{array}{c} 1x1,\;32\\ 3x3,\;64 \end{array}\right]\times 3$	$\left[\begin{array}{c} 1x1,\;32\\ 3x3,\;64 \end{array}\right]\times 2$	$\left[\begin{array}{c} 1x1,\;32\\ 3x3,\;64 \end{array}\right]\times 2$	$\left[\begin{array}{c} 1x1,\;32\\ 3x3,\;64 \end{array}\right]\times 2$	$\left[\begin{array}{c} 1x1,\;32\\ 3x3,\;64 \end{array}\right]\times 2$
CNN Block3	$\left[\begin{array}{c} 1x1,\;64\\ 3x3,\;128 \end{array}\right]\times 4$	$\left[\begin{array}{c} 1x1,\;64\\ 3x3,\;128 \end{array}\right]\times 4$	$\left[\begin{array}{c} 1x1,\;64\\ 3x3,\;128 \end{array}\right]\times 4$	$\left[\begin{array}{c} 1x1,\;64\\ 3x3,\;128 \end{array}\right]\times 4$	$\left[\begin{array}{c} 1x1,\;64\\ 3x3,\;128 \end{array}\right]\times 4$	$\left[\begin{array}{c} 1x1,\;64\\ 3x3,\;128 \end{array}\right]\times 4$	$\left[\begin{array}{c} 1x1,\;64\\ 3x3,\;128 \end{array}\right]\times 2$	$\left[\begin{array}{c} 1x1,\;64\\ 3x3,\;128 \end{array}\right]\times 3$	$\left[\begin{array}{c} 1x1,\;64\\ 3x3,\;128 \end{array}\right]\times 4$	$\left[\begin{array}{c} 1x1,\;64\\ 3x3,\;128 \end{array}\right]\times 4$
CNN Block4	$\left[\begin{array}{c} 1x1,\;128\\ 3x3,\;256 \end{array}\right]\times 4$	$\left[\begin{array}{c} 1x1,\;128\\ 3x3,\;256 \end{array}\right]\times 4$	$\left[\begin{array}{c} 1x1,\;128\\ 3x3,\;256 \end{array}\right]\times 5$	$\left[\begin{array}{c} 1x1,\;128\\ 3x3,\;256 \end{array}\right]\times 6$	$\left[\begin{array}{c} 1x1,\;128\\ 3x3,\;256 \end{array}\right]\times 4$	$\left[\begin{array}{c} 1x1,\;128\\ 3x3,\;256 \end{array}\right]\times 4$	$\left[\begin{array}{c} 1x1,\;128\\ 3x3,\;256 \end{array}\right]\times 3$	$\left[\begin{array}{c} 1x1,\;128\\ 3x3,\;256 \end{array}\right]\times 3$	$\left[\begin{array}{c} 1x1,\;128\\ 3x3,\;256 \end{array}\right]\times 4$	$\left[\begin{array}{c} 1x1,\;128\\ 3x3,\;256 \end{array}\right]\times 4$
CNN Block5	$\left[\begin{array}{c} 1x1,\;256\\ 3x3,512 \end{array}\right]\times 5$	$\left[\begin{array}{c} 1x1,\;256\\ 3x3,512 \end{array}\right]\times 5$	$\left[\begin{array}{c} 1x1,\;256\\ 3x3,512 \end{array}\right]\times 6$	$\left[\begin{array}{c} 1x1,\;256\\ 3x3,512 \end{array}\right]\times 4$	$\left[\begin{array}{c} 1x1,\;256\\ 3x3,512 \end{array}\right]\times 9$	$\left[\begin{array}{c} 1x1,\;256\\ 3x3,512 \end{array}\right]\times 5$	$\left[\begin{array}{c} 1x1,\;256\\ 3x3,512 \end{array}\right]\times 7$	$\left[\begin{array}{c} 1x1,\;256\\ 3x3,512 \end{array}\right]\times 7$	$\left[\begin{array}{c} 1x1,\;256\\ 3x3,512 \end{array}\right]\times 9$	$\left[\begin{array}{c} 1x1,\;256\\ 3x3,512 \end{array}\right]\times 10$
CNN Block6	$\left[\begin{array}{c} 1x1,\;256\\ 3x3,512 \end{array}\right]\times 8$	$\left[\begin{array}{c} 1x1,\;256\\ 3x3,512 \end{array}\right]\times 9$	$\left[\begin{array}{c} 1x1,\;256\\ 3x3,512 \end{array}\right]\times 9$	$\left[\begin{array}{c} 1x1,\;256\\ 3x3,512 \end{array}\right]\times 8$	$\left[\begin{array}{c} 1x1,\;256\\ 3x3,512 \end{array}\right]\times 5$	$\left[\begin{array}{c} 1x1,\;256\\ 3x3,512 \end{array}\right]\times 8$	-	$\left[\begin{array}{c} 1x1,\;256\\ 3x3,1024 \end{array}\right]\times 3$	$\left[\begin{array}{c} 1x1,\;256\\ 3x3,512 \end{array}\right]\times 5$	$\left[\begin{array}{c} 1x1,\;256\\ 3x3,512 \end{array}\right]\times 6$
Dense1	512	512	512	512	512	512	512	512	512	512
	GAP	GAP	GAP	GAP	GAP	GAP	GAP	GAP	GAP	GAP
Dropout	0.4	0.4	0.4	0.3	0.4	0.4	0.4	0.4	0.4	0.4
Dense2	128	128	128	128	128	128	128	128	128	128

**Table 6:** Performance metrics of the transfer learning models.

Model	Accuracy (%)	Precision (%)	Recall (%)	F1-Score
DenseNet121	92.35	90.89	94.27	92.32
DenseNet169	93.49	92.17	93.96	92.95
DenseNet201	93.11	91.60	94.57	92.88
EfficientNetV2B0	85.27	83.35	87.14	84.66
EfficientNetV2B3	91.21	89.83	90.44	89.98
EfficientNetV2M	91.72	90.13	93.31	91.52
EfficientNetV2S	90.27	89.14	90.11	89.47
InceptionV3	89.38	87.32	89.22	87.99
InceptionResNetV2	91.40	89.88	91.43	90.52
MobileNetV2	90.77	88.58	91.00	89.54
RegNetX008	93.68	92.04	94.55	93.19
RegNetY008	93.11	91.42	93.72	92.42
ResNet50	90.90	89.07	92.20	90.34
ResNet101	92.04	90.96	90.63	90.74
ResNet152	91.15	90.38	90.21	90.20
ResNetRS50	92.86	90.76	93.15	91.85
ResNetRS100	91.97	91.02	91.69	91.20
VGG16	81.73	77.34	80.64	78.61
VGG19	89.13	86.76	89.82	87.93
Xception	92.79	91.87	92.48	92.10

**Table 7:** Performance metrics of the custom models that contain CNN layers and residual connections.

Model	Accuracy (%)	Precision (%)	Recall (%)	F1 Score
C1	87.73	84.68	87.22	85.69
C2	91.91	89.72	92.70	91.07
C3	91.02	88.84	91.61	89.97
C4	91.40	89.51	92.28	90.68
C5	92.41	90.25	93.51	91.61
C6	92.79	90.90	93.89	92.20
C7	92.98	91.71	93.27	92.33
C8	92.04	90.21	91.70	90.85
C9	92.41	91.10	92.92	91.79
C10	93.62	92.32	94.36	93.19

**Table 8:** Performance metrics of the custom models that contain attention modules.

Model	Accuracy (%)	Precision (%)	Recall (%)	F1-Score
C11	93.05	91.76	93.44	92.47
C12	93.81	93.13	93.46	93.28
C13	92.16	90.60	92.76	91.50
C14	93.68	92.71	94.40	93.34
C15	94.06	93.21	94.86	93.90
C16	94.18	92.69	95.11	93.76
C17	93.43	91.84	94.19	92.80
C18	94.31	92.92	95.28	93.97
C19	94.37	93.26	94.77	93.91
C20	94.69	93.43	95.48	94.33

**Table 9:** Filter sizes and filter numbers in the convolution layers of the MultiHisNet’s blocks.

Block	Filters (Size, Number)
Block A	$\left[3x3,\;32\right]$
Block B	$\left[\begin{array}{c} 1x1,\;32\\ 3x3,\;64 \end{array}\right]$
Block C	$\left[\begin{array}{c} 1x1,\;64\\ 3x3,\;128 \end{array}\right]$
Block D	$\left[\begin{array}{c} 1x1,\;128\\ 3x3,\;256 \end{array}\right]$
Block E	$\left[\begin{array}{c} 1x1,\;256\\ 3x3,512 \end{array}\right]$

**Table 10:** Models for ensemble learning.

Type of Model	Model (Accuracy)	Abbreviation
Best Transfer Learning Models	DenseNet169 (93.49%)	T2
	RegNetX008 (93.68%)	T1
Best Custom Models	C19 (94.37%)	C2
	MultiHisNet (94.69%)	C1

**Table 11:** Results of ensemble models.

Ensemble Model	Models	Weights	Accuracy (%)	Precision (%)	Recall (%)	F1-Score (%)
E1	C1, C2, T2	0.31, 0.20, 0.49	96.46	96.92	96.46	96.58
E2	C1, C2, T1	0.32, 0.29, 0.39	96.46	96.87	96.46	96.57
E3	C2, T1, T2	0.41, 0.38, 0.21	96.52	96.92	96.52	96.63
Proposed Ensemble Model	C1, T1, T2	0.50, 0.18, 0.32	96.71	97.07	96.71	96.81

**Table 12:** Comparison of the custom models.

CNN	Residual Connection	Channel and Spatial Attention Module	Ensemble	Best Performance (Accuracy-%)
√				92.98
√	√			93.62
√	√	√		94.69
√	√	√	√	96.71

**Table 13:** Comparison with similar studies using MI multiclass classification on the BreakHis dataset.

Research	Model	Classes	Dataset Split	Data Augmentation	Accuracy (%)
Boumaraf et al. [11]	Transfer Learning (ResNet-18)	8	80–20	Yes	92.03
Yari et al. [15]	Transfer Learning (ResNet-50)	8	75–20−5	Yes	94.33
Vikranth et al. [13]	Transfer Learning (DenseNet201, ResNet50, and MobileNetV2) ^1^	8	-	Yes	92
Zaalouk et al. [16]	Transfer Learning (Xception, DenseNet201, InceptionResNetV2, VGG19, and ResNet152) ^2^	8	70–20−10	Yes	93.32
Umer et al. [21]	Ensemble of features	8	70–30	-	90.1
Tummala et al. [25]	Ensemble of SwinT	8	70–30	No	93.4
Wang et al. [46]	Ensemble of transfer learning models	8	-	Yes	94.11
Proposed Ensemble Model	Ensemble of custom and transfer learning models	8	80–20	Yes	96.71

^1^ Among the models included in the study by Vikranth et al. [13], the best-performing model is MobileNetV2, and its results are presented in the table. ^2^ Among the models studied by Zaalouk et al. [16], the best-performing model is Xception, and its results are presented in the table.

## Data Availability

Data derived from public domain resources. The data presented in this study are available in The Laboratory of Vision, Robotics and Imaging (VRI) available online: https://web.inf.ufpr.br/vri/databases/breast-cancer-histopathological-database-breakhis/ (accessed on 22 January 2024).
